# Ferritin Heavy-like subunit is involved in the innate immune defense of the red swamp crayfish *Procambarus clarkii*

**DOI:** 10.3389/fimmu.2024.1411936

**Published:** 2024-07-23

**Authors:** Si-Pei Zhang, Jie Zhang, Qing-Hao Wang, Yang Ye, Dai-Zhen Zhang, Qiu-Ning Liu, Bo-Ping Tang, Li-Shang Dai

**Affiliations:** ^1^ Jiangsu Key Laboratory for Bioresources of Saline Soils, Jiangsu Synthetic Innovation Center for Coastal Bio-agriculture, Jiangsu Provincial Key Laboratory of Coastal Wetland Bioresources and Environmental Protection, School of Wetlands, Yancheng Teachers University, Yancheng, China; ^2^ School of Pharmaceutical Sciences, Wenzhou Medical University, Wenzhou, China

**Keywords:** *Procambarus clarkii*, Ferritin Heavy-like subunit, RNAi, gene expression, immune response

## Abstract

Iron-binding proteins, known as ferritins, play pivotal roles in immunological response, detoxification, and iron storage. Despite their significance to organisms, little is known about how they affect the immunological system of the red swamp crayfish (*Procambarus clarkii*). In our previous research, one ferritin subunit was completely discovered as an H-like subunit (*PcFeH*) from *P. clarkii*. The full-length cDNA of *PcFerH* is 1779 bp, including a 5’-UTR (untranslated region, UTR) of 89 bp, 3’-UTR (untranslated region, UTR) of 1180 bp and an ORF (open reading frame, ORF) of 510 bp encoding a polypeptide of 169 amino acids that contains a signal peptide and a Ferritin domain. The deduced PcFerH protein sequence has highly identity with other crayfish. PcFerH protein’s estimated tertiary structure is quite comparable to animal structure. The *PcFerH* is close to *Cherax quadricarinatus*, according to phylogenetic analysis. All the organs examined showed widespread expression of *PcFerH* mRNA, with the ovary exhibiting the highest levels of expression. Additionally, in crayfish muscles, intestines, and gills, the mRNA transcript of *PcFerH* was noticeably up-regulated, after LPS and Poly I:C challenge. The expression of downstream genes in the immunological signaling system was suppressed when the *PcFerH* gene was knocked down. All of these findings suggested that *PcFerH* played a vital role in regulating the expression of downstream effectors in the immunological signaling pathway of crayfish.

## 1 Introduction

*Procambarus clarkii* is a species of freshwater crayfish that is indigenous to southern and southeastern United States, southern Mexico, and northern Mexico, commonly known as crayfish (1). Due to its great commercial value and delectability, crayfish, known scientifically as *Procambarus clarkii*, has recently become the most prominent freshwater crustacean species in inland China. It is a plentiful source of premium proteins and includes all the essential amino acids for human nutrition (2, 3). Unfortunately, catastrophic infectious disease outbreaks brought on by parasites, bacteria, and viruses, such as white spot syndrome virus, *Vibrio parahaemolyticus*, *Vibrio harveyi*, have put *P. clarkii* aquaculture in danger and resulted in considerable economic losses (4). Moreover, *P. clarkii* is used as an invertebrate model organism for research on the molecular mechanisms underlying the innate immune system (5). Consequently, studying the innate immune system of the model organism *P. clarkii* can help further investigate the systems of invertebrates. Investigating *P. clarkii’*s innate immune mechanisms also can lead to the creation of fresh approaches to the management and prevention of illness.

The only line of protection against foreign antigens and invasive infections is the innate immune system of crustaceans (6), which is separated into humoral and cellular defensive responses (7). The generation of reactive oxygen and nitrogen intermediates, antimicrobial peptides, hemolymph coagulation and melanization cascades, and antimicrobial peptides are all elements of humoral defenses (8–10). Hemocyte-mediated reactions like phagocytosis and encapsulation are examples of cellular defenses (11, 12). Hemocytes, on the other hand, are the main players in cellular immunity; they have the ability to recognize a range of foreign targets against a variety of infectious illnesses with alterations to self, ensuring successful defensive responses (13). Poly I:C is a kind of synthetic double-stranded RNA (dsRNA), because of its structure is similar to a variety of ribonucleic acid produced by virus in cell metabolism, are often used to analog immune stimulation of virus infection. LPS is the outermost structure of the cell wall of Gram-negative bacteria. It is a complex of lipids, polysaccharides and proteins (14). qPCR was used to analyze the expression changes of PcFerH under the stimulation of Poly I:C and LPS, and to provide basic data for further exploring the molecular response pattern of PcFerH in the process of resistance to pathogen invasion.

Many living things, including microbes, plants, vertebrates, and invertebrates, have ferritin, which they all share in common (15, 16). A 24 subunit spherical protein with a light (FTL) and heavy chain (FTH1) subunit mix makes up ferritin (17, 18). Moreover, ferritin, which is the main protein for storing iron in most living things, participates in a variety of biological activities, containing cell stimulation, antioxidation, angiogenesis, removal of certain heavy metal toxicity, immunological response, and maintenance of iron metabolic balance (19, 20). The ferritin gene is controlled both transcriptionally and post-transcriptionally (21), and iron response proteins mediates the latter regulation (22). By its distinctive iron withholding capacity, ferritins have been shown to play a role in innate immunity in both vertebrate and invertebrate organisms (23, 24). At present, Ferritin has been cloned and characterized from *P. clarkii* (25). And its expression patterns under different tissues and immune stress were studied separately. These data provide new insights into the physiological role of ferritin. However, whether it participates in the innate immunity of crayfish and what role it plays in the innate immunity of crayfish is unknown.

In summary, this study, by means of immune stimulation and RNA interference, had elucidated the expression of ferritin in various tissues of crayfish; the changes in the expression of ferritin in hepatopancreas, muscles, intestines, and gills after LPS and Poly I:C immune challenges; and the impact of the PcFer dsRNA (dsPcFer) on Toll and Imd signal pathway related genes.

## 2 Materials and methods

### 2.1 *P. clarkii* and tissue collection

*P. clarkii* were bought at the aquatic market in Yancheng, Jiangsu Province, China, weighing 20–25 grams apiece. The crayfish were raised for two weeks in a laboratory incubator supplied with fresh water at 24°C prior to the experiment (26). The crayfish was dissected for its hepatopancreas, muscles, intestines, gills, hearts, stomachs, ovaries, spermaries, brains, ventral nerve cords and antennal glands. Five crayfish were used to extract the hemolymph, and right away, blood cells were separated by centrifugation at 2000 rpm for 10 minutes at 4°C (26) after adding an equivalent amount of anticoagulant. All tissues, with the exception of hemolymph, were frozen in liquid nitrogen to be used for gene expression analysis.

### 2.2 Immunity challenge

On average, three groups of crayfish were formed through random division. Each crayfish had its abdominal segment injected with 100 uL LPS or Poly I:C for the immune challenge. The control received an identical amount of PBS injection (27). Five crayfish hepatopancreas, muscles, intestines, and gills were randomly sampled for quantitative real-time PCR at 12, 24, 36, and 48 hours after injection (qRT-PCR) (28).

### 2.3 RNA extraction and cDNA synthesis

Using RNA isolater Total RNA Extraction Reagent, total RNA was extracted from various crayfish tissues in the control or challenge groups at various time points (Vazyme, China). They were then dispersed in DEPC-treated water. A 1% agarose gel electrophoresis without RNase was used to evaluate the quality of the RNA. The cDNA Synthesis Kit was then used to create the first strand of cDNA (Vazyme, China). In order to conduct further research, the cDNA samples were finally stored in a -80°C cryogenic refrigerator.

### 2.4 Cloning of FerH gene

Ferritin Heavy-like subunit was named *FerH* gene in this study. Previous transcriptome data were used to determine the precise forward and reverse primers for the *FerH* gene. The following PCR techniques were used in conjunction with the Advantage 2 PCR Kit (Clontech, USA) to successfully clone the complete length of the Ferritin H-like subunit: 95°C for 3 min; twenty-five cycles at 95°C for 20 s, 55°C for 25 s and 72°C for 30 s; 72°C for 10 min. The PCR products were in-gel purified using a gel purification kit from Vazyme in China before being ligated onto a pMD-18T vector from Takara and subsequently transformed into competent DH5α cells. The positive recombinants were found using the blue-white color selection in ampicillin-containing LB plates and the PCR screening using the two different primers (F1 and R1), respectively. Positive clones were sequenced by Sangon company (Shanghai, China) (29).

### 2.5 Sequence blast and phylogenic analysis for FerH gene

The online Expert Protein Analysis System (http://www.expasy.org) was used to gather the deduced amino acid sequences, and an open reading frame (ORF) finding tool (http://ncbi.nlm.nih.gov/gorf/gorf.html) was used to assess them. Potential domains were anticipated using the Simple Modular Architecture Research Tool program (http://smart.embl-heidelberg.de). In order to determine if the dataset is suitable for building trees, homologous sequences from various species were compared using MAFFT, Gblocks was employed to eliminate sections of the alignments deemed unreliable. Subsequently, MEGA version 6 was used to build a phylogenetic tree, applying the Neighbor-Joining (NJ) approach, with a bootstrap validation consisting of 1000 iterations (28).

### 2.6 Quantitative real-time PCR analysis for expression patterns

The first strand of cDNA was reverse-transcribed using RNA from each tissue after total RNA was collected from various tissues of unchallenged crayfish or challenged crayfish at various time periods. The genespecific primers were designed. To serve as an internal control, Pc18S was increased. The qRT-PCR was conducted in a total volume of 10 liters, which included 5 liters of qPCR Mix (Vazyme, China), 2 liters of cDNA that had been diluted 1:9, 0.5 liters for each forward primer and reverse primer, and 2 liters of ddH_2_O. The following was the PCR amplification process: 95°C for 2min, 40 cycles of 95°C for 15s, 55°C for 20 s, 72°C for30 s, and a melting curve analysis from 60–95°C. Moreover, relative expression values that were computed using 2^−ΔΔCT^, were used to display the expression level (30).

### 2.7 Double strand RNA synthesis and RNAi assay

The double-stranded RNA (dsRNA) targeting the *PcFerH* or GFP (as control) was synthesized with an *in vitro* transcription T7 kit (Vazyme, China). Using the gene-specific primers ds-PcFerH-F and ds-PcFerH-R or ds-GFP-F and ds-GFP-R, PCR was used to create the DNA template for the synthesis of PcFer-dsRNA or GFP-dsRNA (**Table 1**). The dsRNA synthesis system, which had a total volume of 20 μl, was made using the following methods: 8 μg of DNA templates, 2 μl of transcription buffer, 8 μl of NTP mix, 2 μl of T7 enzyme mix, and 20 μl of RNase-Free water. The mixed samples were tested in the PCR for 2 hours at 37°C. The solution was then added, bringing the total volume to 40 μl, along with 1 μl DNase I, 17 μl RNase-freewater and 2 μl RNase T1(10 U/μl). The mixed solution was then re-incubated for 30 minutes at 37°C. The RNA product was then cleaned using a magnetic bead technique.

**Table 1:** The primers used in this study.

Primer name	Primer sequences (5′-3′)
*PcFerH-RC5*	GAATATCCTGCAGGACAACACGA
*PcFerH-RC3*	AGACATGGTGACAAAGCACCA
*Pc18S-F*	ACCGATTGAATGATTTAGTGAG
*Pc18S-R*	TACGGAAACCTTGTTACGAC
*PcFerH-Fi*	GCGTAATACGACTCACTATAGGGTGCTATCCGCCAAAACTACC
*PcFerH-Ri*	GCGTAATACGACTCACTATAGGGACCCTCTGGTGCTTTGTCAC
*GFP-Fi*	GCGTAATACGACTCACTATAGGTGGTCCCAATTCTCGTGGAAC
*GFP-Ri*	GCGTAATACGACTCACTATAGGCTTGAAGTTGACCTTGATGCC
*PcFerH-RT-F*	CAGCGTGGAGGTCGTGTTGT
*PcFerH-RT-R*	CTCGCCTAAGAAATTACCATTCAG
*PcALF9-RT-F*	GTCGGGCTGTTTAGGAATGAGG
*PcALF9-RT-R*	TTGTCTTGTTCGCCACTCCACTT
*PcALF10-RT-F*	TGTCTGCTCTTTGCTCGTTC
*PcALF10-RT-R*	GTGTCGTCAATAGATACTGCGTTA
*Pccrustin2-RT-F*	CTGGTGTTGTCCATGCTGGTG
*Pccrustin2-RT-R*	CCTGAGGTGGTAGGATTCTTGT
*Pclysozyme-RT-F*	AGCCCTCGTGGTCGTCTTG
*Pclysozyme-RT-R*	GTTGGGATCGGCGTTATTG
*PcRelish-RT-F*	GCTGTCCGTGGCAATGAAG
*PcRelish-RT-R*	GAGGCAGTGCTGAACGAGTG

Crayfish were randomly separated into two groups, one receiving ds-PcFerH injections and the other receiving ds-GFP injections. Each crayfish was given an injection of the prepared dsRNA into the abdomen region (28). The experimental group consisted of crayfish that had received 10 μg of PcFerH-dsRNA injections, and the control group received 10 μg of GFP-dsRNA injections. Five people’s hepatopancreas, muscles, intestines, and gills were taken after 24 hours in order to extract total RNA. The remaining crayfish were separated into three groups at the same time, with one group receiving injections of 100 liters of LPS (0.1 μg/g), another 100 liters of Poly I:C (1 μg/g), and the third group receiving an equivalent volume of PBS as a control. After 24 hours, five different individuals’ hepatopancreas, muscles, intestines, and gills were similarly taken to extract total RNA. qRT-PCR was performed using the primers *Pc-PcFerH-qRT-F* and *Pc-PcFerH-qRT-R* to assess the knockdown effectiveness of the ferritin heavy-like gene.

### 2.8 Detection of innate immunity signaling pathway associated genes

The mRNA expression levels of the genes connected to the Toll and Imd signaling pathways in the aforementioned RNAi test were detected using qRT-PCR in order to examine the function of the *Ferritin Heavy-like* gene in the crayfish innate immune system (31). Specifically, LPS, Poly I:C, and PBS were each injected into the abdomen region of each crayfish in each group after 24 hours of dsRNA treatment, along with the Ferritin Heavy-like gene dsRNA (dsPcFerH) injection group and the GFP dsRNA (dsGFP) control group. After that, five individuals’ hepatopancreas, muscles, intestines, and gills were removed from three groups of crayfish 24 hours following an immune challenge. The whole RNA was then extracted, and cDNA was produced. Additionally, qRT-PCR was used to find the mRNA expression levels of the related genes. In **Table 1**, a set of primers for qRT-PCR were displayed.

### 2.9 Statistical analysis

Three separate experiments’ worth of numerical data were combined and subjected to one-way analysis of variance (ANOVA), and the results were then subjected to a Student’s *t*-test. Differences with *p* < 0.05 were considered as significant difference (32).

## 3 Results

### 3.1 Sequence analysis of the FerH gene

**Figure 1:**
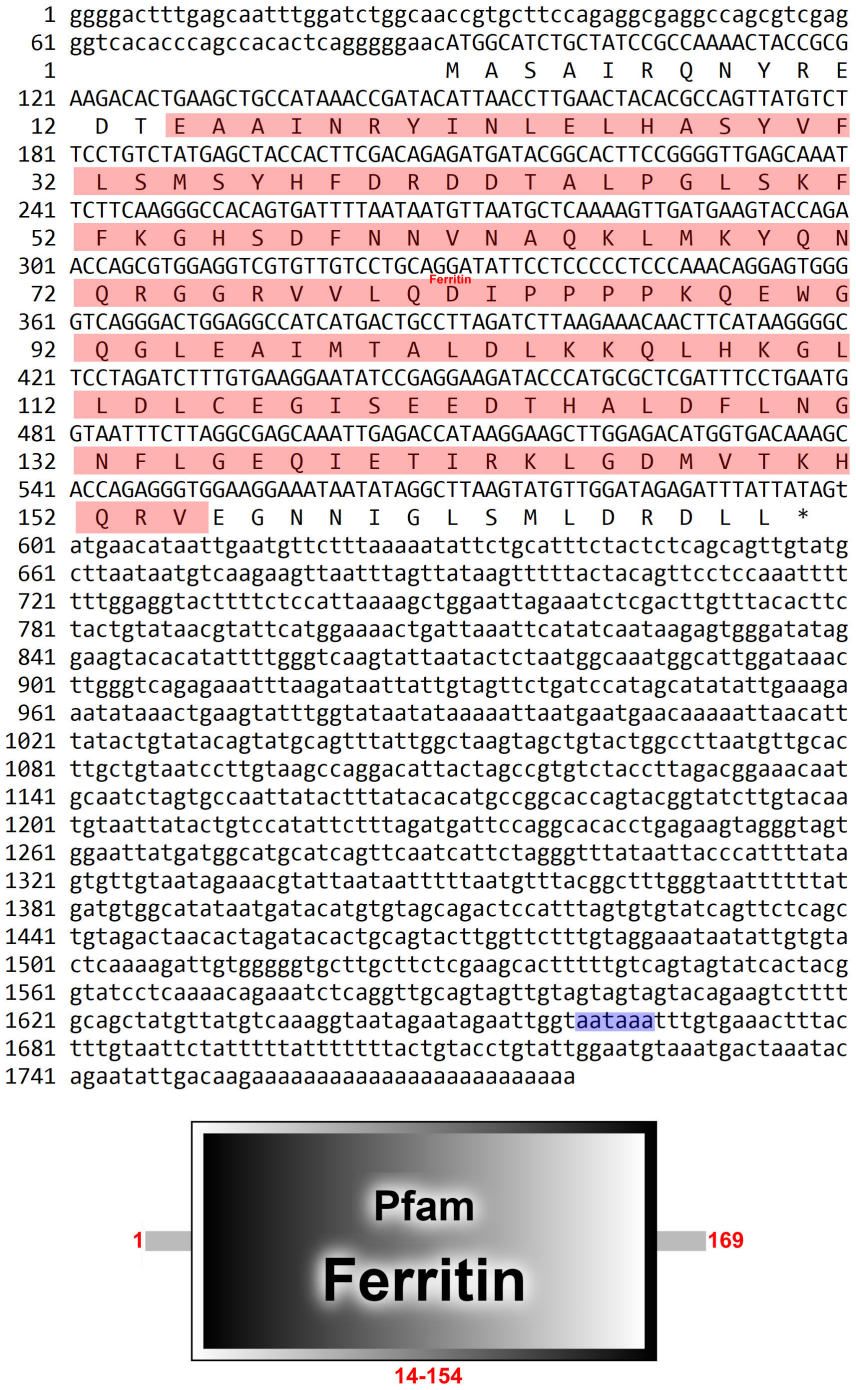
Nucleotides and deduced amino acid sequence of PcFerH gene. The red shadow represented the functional domain. And blue shadow showed termination signal.

In our earlier transcriptome test, a fragment of 1,720 bp cDNA was obtained by PCR from *P. clarkii*’s hepatopancreas. BLAST online analysis revealed that the *FerH* gene’s open reading frame (ORF) contained 150 amino acids, with a functional domain having 122 amino acid residues (**Figure 1**).

### 3.2 Homologous alignment and phylogenetic analysis

**Figure 2:**
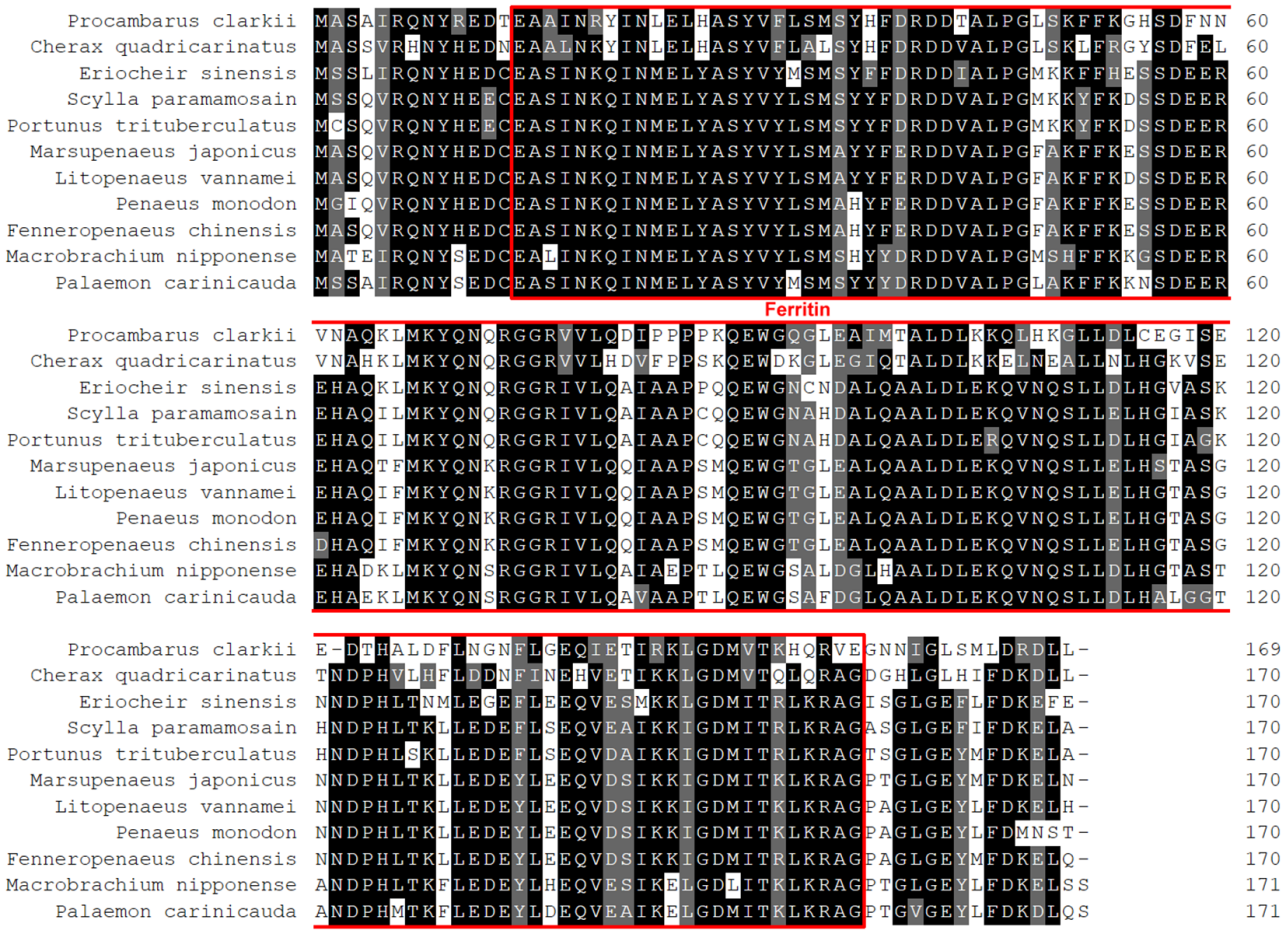
Multiple alignments of PcFerH gene. The number on the right standed for the locations of the amino acids in various sequences. The red border indicated the functional domain of PcFerH gene. The distinct amino acid conservations are shown by various colors.

**Figure 3:**
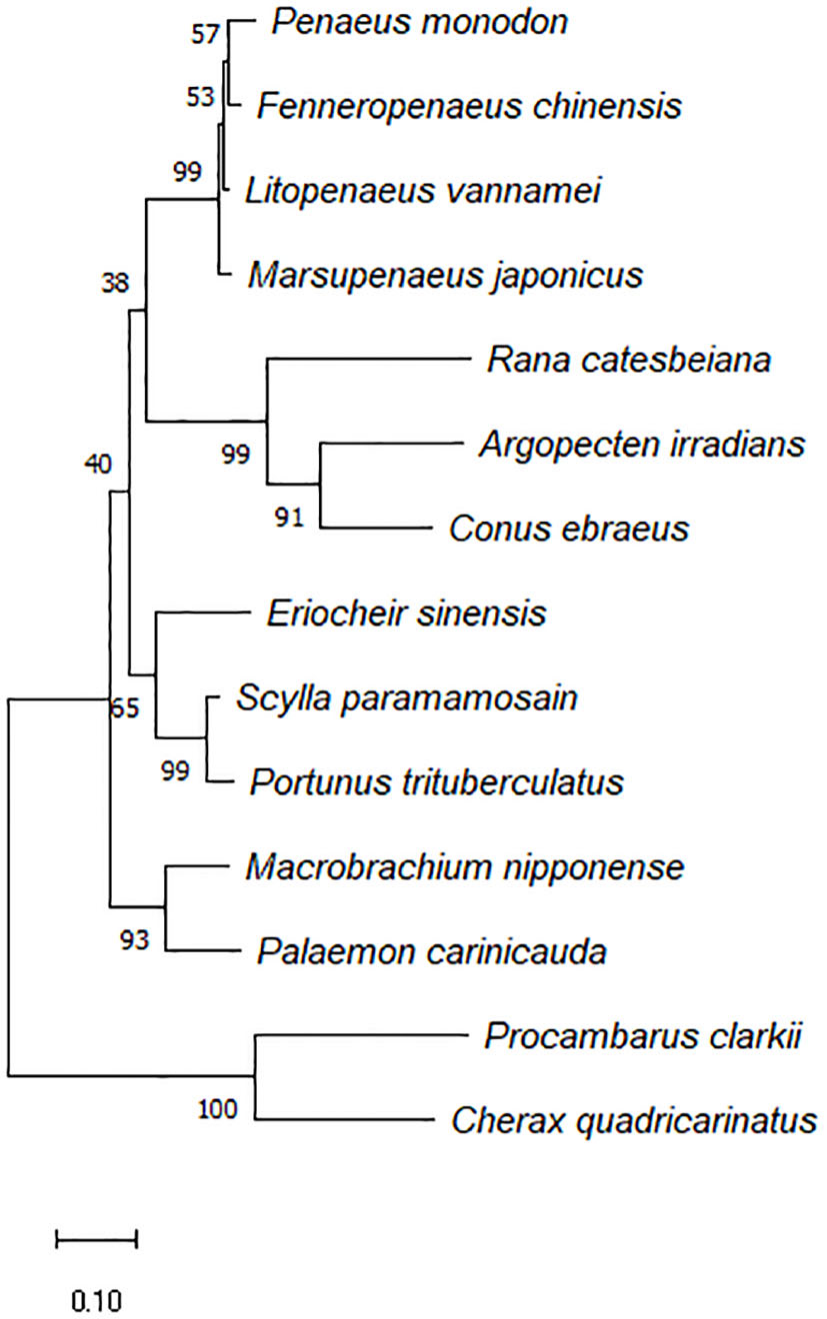
Phylogenetic tree constructed using the inferred amino acid sequences of several animal PcFerH gen, phylogenetic tree builted with MEGA 6.06 and the neighbor-joining technique based on ferritin amino acid sequences. The branches’ bootstrap percentages (based on 1000 replications) were shown.

**Figure 2** depicted a comparison between the crayfish and its related species. The results demonstrated that crawfish and its related species had high homology in functional domain and have conserved sequence. And the phylogenetic relationship between crayfish and its related species of *FerH* gene was revealed in **Figure 3**. It was found that *P. clarkii*’s *FerH* gene and *Cherax quadricarinatus’*s *FerH* gene have the closest phylogenetic relationship.

### 3.3 Tissue distribution of the *FerH* gene

**Figure 4:**
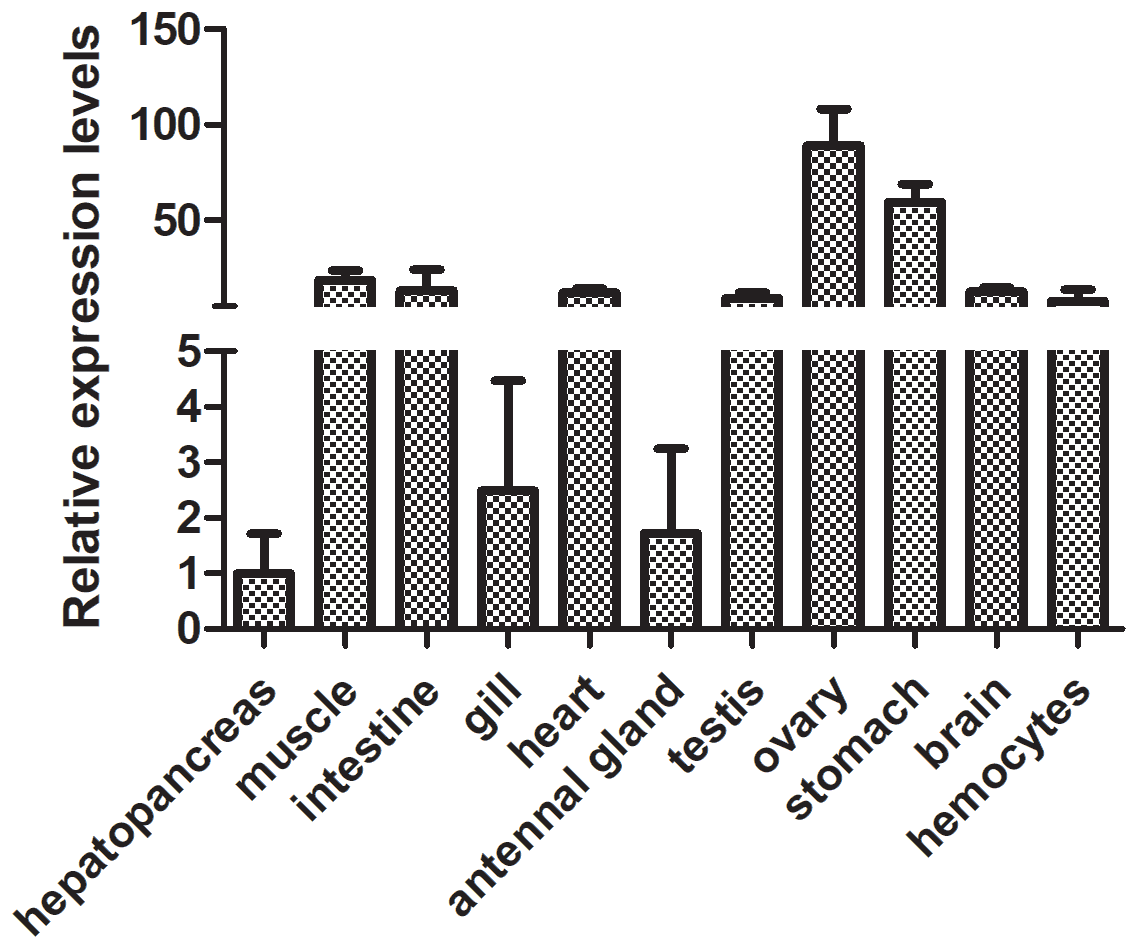
Analysis of the expression patterns of the PcFerH gene in several organs from the normal crayfish, including the hepatopancreas, muscle, intestine, gill, heart, antennal gland, testis, ovary, stomach, brain, and hemocytes, was done using the 18S RNA gene as the inner control.

To ascertain the *FerH* expression profile in several tissues, including the hepatopancreas, muscle, intestine, gill, heart, antennal gland, testis, ovary, stomach, brain, and hemocytes, we used quantitative PCR (qPCR). The study showed that *FerH* mRNA was consistently expressed in all of the tissues that were investigated, with the ovary exhibiting the greatest levels of *FerH* mRNA expression (**Figure 4**).

### 3.4 Quantitative analysis of FerH mRNA after immunity challenge

**Figure 5:**
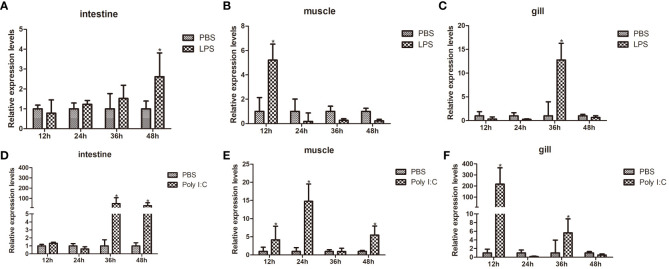
Time course expression patterns of PcFerH gene analyzed by qRT-PCR. **(A-C)** represented the expression profiles in intestines, muscles, and gills at different time points after LPS challenge. **(D-F)** represented the expression profiles in intestines, muscles, and gills at different time points after challenge with Poly I:C. All experimental groups were treated with PBS-injection group as the control group. And 18S RNA gene was used as the inner control. The as-terisks indicated significant differences (*P < 0.05) from the control.

Using qPCR, the transcription levels of *FerH* in the intestines, muscle, and gill were examined at various time periods in order to ascertain the transcriptional expression patterns of *FerH* in response to LPS and Poly I:C challenge. Compared with the control group, after LPS challenge, the FerH transcripts in the intestine were significantly upregulated at 48 hours (**Figure 5A**). The highest level of *FerH* mRNA in muscles was detected at 12 hours (**Figure 5B**). The FerH transcript in the gills was significantly upregulated and reached its peak at 36 hours (**Figure 5C**). After challenge with Poly I: C, the *FerH* transcripts in the intestine were significantly upregulated at 36 and 48 hours (**Figure 5D**). The expression level of *FerH* in muscles was significantly upregulated at 12, 24, and 48 hours and reached its peak at 24 hours (**Figure 5E**). The FerH transcripts in the gills were significantly upregulated at 12 and 24 hours and peaked at 12 hours (**Figure 5F**). Although the expression profile of the PBS-injected control group varied modestly over time, no variations that were statistically significant were discovered. All of these findings suggest that *FerH* was crucial for pathogen defense.

### 3.5 FerH affect the transcription of innate immunity signaling pathway associated genes

**Figure 6:**
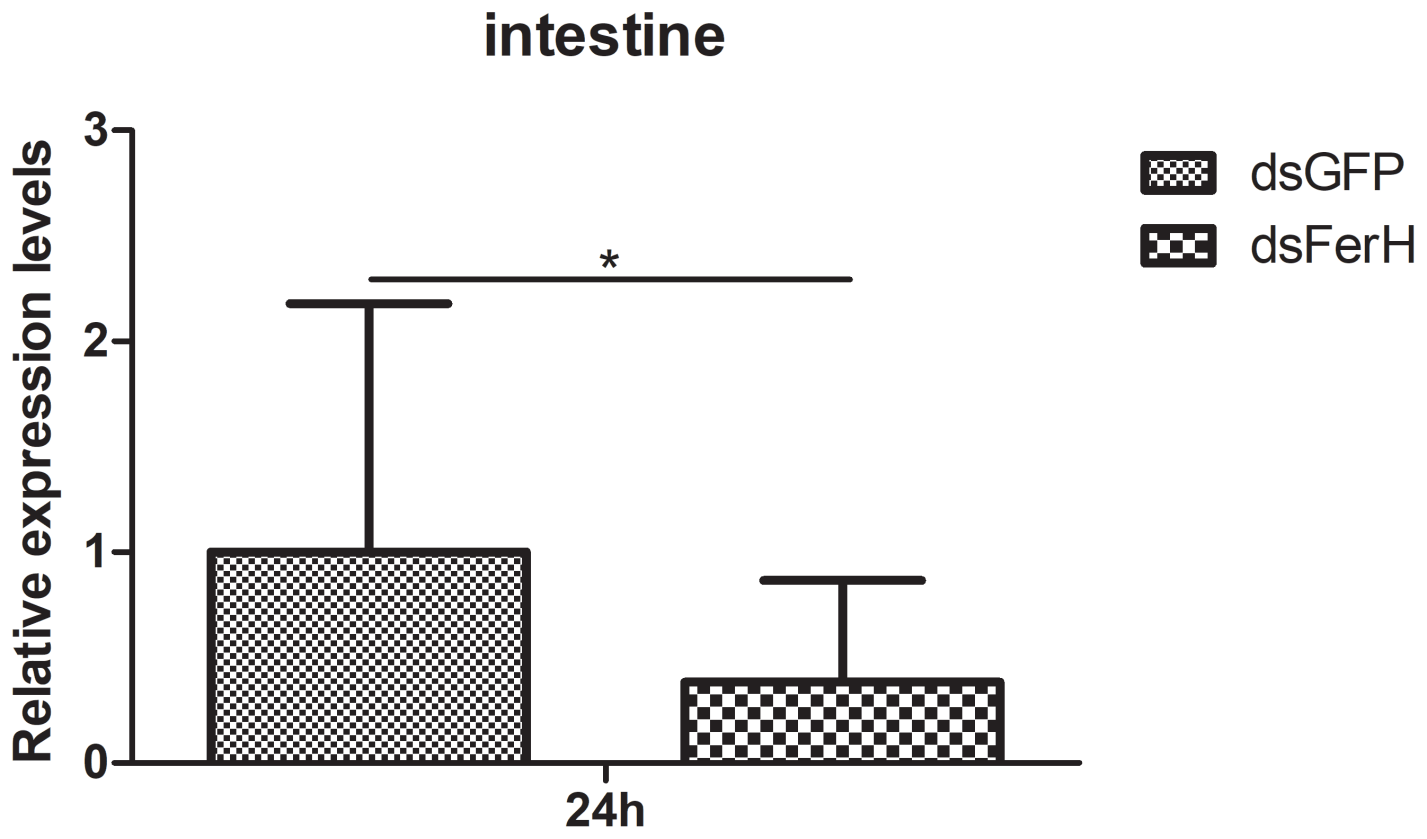
The RNAi experiment was used to verifies that specific siRNA can inhibit the expression of *PcFerH*, qPCR results indicated that crayfish intestines drastically reduce the transcription of the target gene by injecting dsRNA PcFerH. Crayfish injected with dsRNA against GFP as control. The asterisks indicated significant differences (*P < 0.05) from the control.

**Figure 7:**
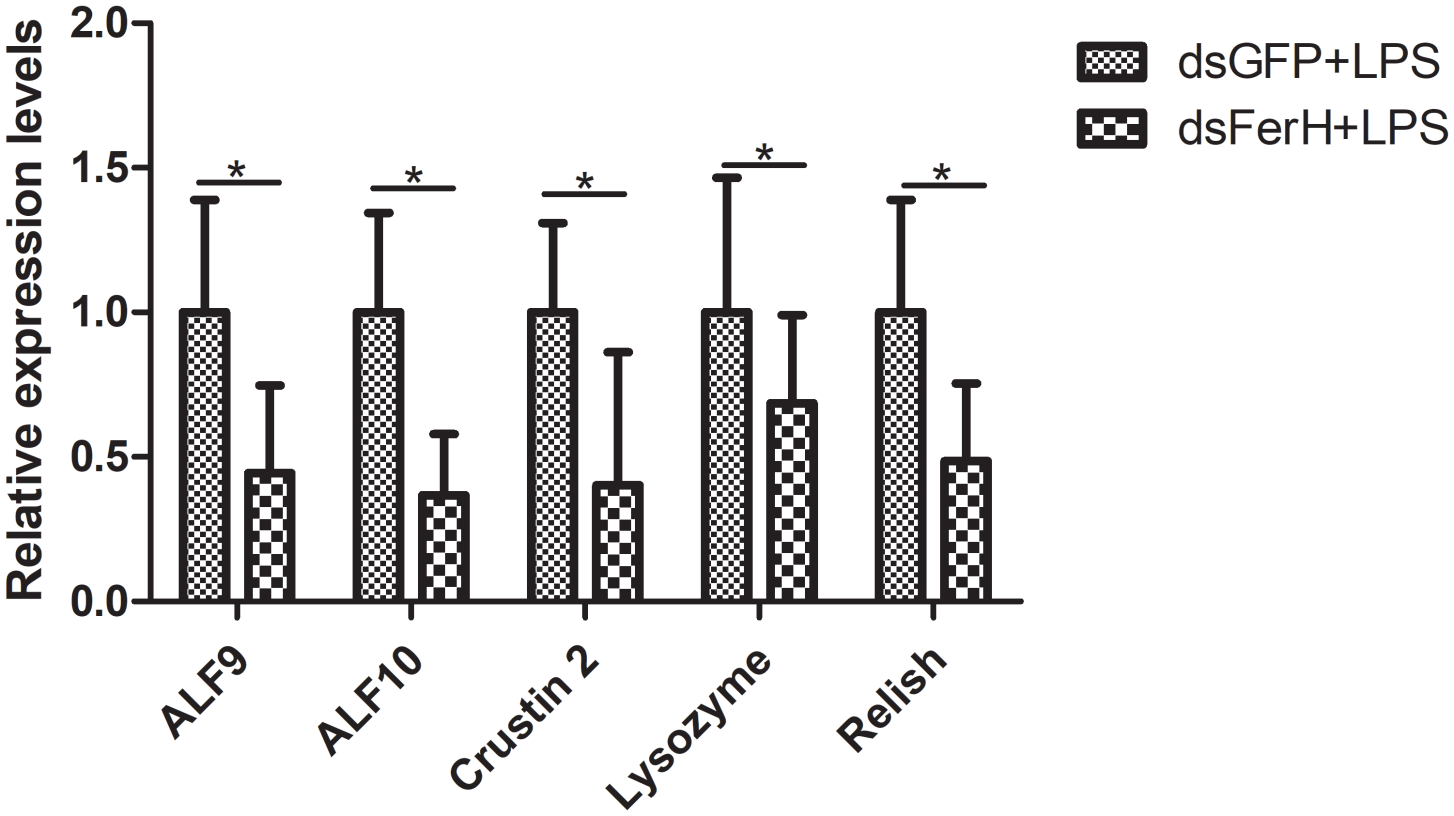
Effect after 24h PcFerH-dsRNA and 24h LPS challenge in the expression of downstream genes in the immunological signaling pathway, including anti-lipopolysaccharide factor 9 (ALF 9), anti-lipopolysaccharide factor 10 (ALF 10), crustin 2 gene (Crustin 2), Lysozyme gene (Lysozyme), and Relish gene (Relish). Significant deviations were shown by asterisks (*p* < 0.05) from control.

**Figure 8:**
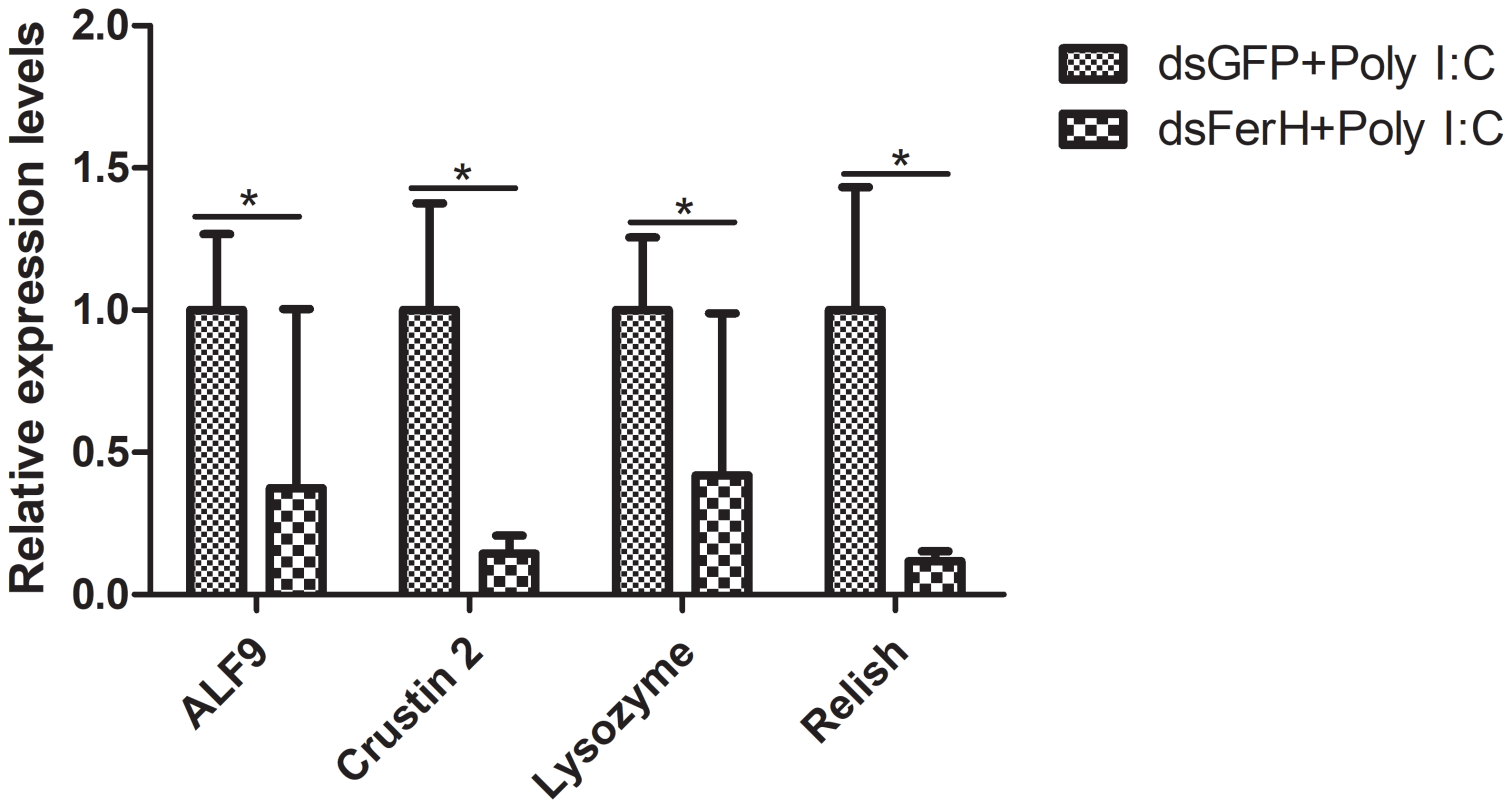
Effect after 24h PcFerH-dsRNA and 24h Poly I: C challenge in the expression of downstream genes in the immunological signaling pathway, including anti-lipopolysaccharide factor 9 (ALF 9), anti-lipopolysaccharide factor 3 (ALF 10), crustin 2 gene (Crustin 2), Lysozyme gene (Lysozyme), and Relish gene (Relish). Significant deviations were shown by asterisks (*p < 0.05) from control.

The RNAi experiment was used to validate the function of the *FerH* gene in the innate immune response of crayfish. Total RNA was collected from the crayfish in each of the three groups’ intestines twenty-four hours after injecting dsRNA in order to measure the FerH gene’s expression level. Results demonstrated that the FerH gene had been successfully knocked down (**Figure 6**). The crayfish were then split into two groups and given separate injections of LPS and Poly I: C. Total RNA was collected from the intestines of the crayfish in each of the three groups in order to determine the level of mRNA expression of downstream genes in the immune signaling pathway using qRT-PCR. This was done in order to further establish the function of the FerH gene in the crayfish innate immunity system. The immune signaling pathway’s downstream genes’ expression levels were compared to those of the group that received dsGFP injections, including anti-lipopolysaccharide factor 9 (ALF 9), anti-lipopolysaccharide factor 3 (ALF 10), crustin 2 gene (Crustin 2), Lysozyme gene (Lysozyme), and Relish gene (Relish), was significantly downregulated after the FerH gene was knocked down, whether stimulated by LPS or Poly I:C (**Figures 7**, **8**). These results revealed that the FerH gene mediates the innate immunity of crayfish, and downregulates the expression of downstream genes in the immune signaling pathway.

## 4 Discussion

The innate immune response mechanism has always been a research hotspot in invertebrates. Among invertebrates, ferritin subunits have been already identified and completely characterized in *Haemaphysalis flava* (33), *Bombyx mori* (34), Lymnaea stagnalis (35), Pinctada fucata (36), Meretrix meretrix (37) and Haliotis rufescens (38), etc. Although ferritin’s primary function is in the metabolism of iron, more recent research has revealed that ferritin also plays a part in cellular immunity (23, 24). The cytosolic ferritins in vertebrates are made up of the Heavy (H) and Light (L) subunit types (39). The H subunit has a catalytic iron binding site, which constitutes the center of iron oxidase. In some cell types, the H ferritin can go to the nucleus to shield DNA from toxicity, or it can be actively released, performing a variety of tasks (39). There is currently little research on the role of H ferritin in the innate immune process of invertebrates. Crawfish serve as a model organism for the study of invertebrates’ innate immune systems. Therefore, the primary emphasis of our study is on the role of H-ferritin in crayfish immunological function.

In this study, *Ferritin Heavy-like subunit* was named *FerH* gene. The full-length cDNA of *FerH* gene is 1720 bp (**Figure 1**). Results of sequence alignment implied that crawfish and its related species have high homology in functional domain and have conserved sequence (**Figure 2**). Results of phylogenetic analysis implied that *Procambarus clarkii*’s *FerH* gene and *Cherax quadricarinatus’*s *FerH* gene have the closest phylogenetic relationship (**Figure 3**).

By using a qRT-PCR experiment, expression patterns of the target gene were discovered, further confirming the function of the *FerH* gene in the innate immune response of crayfish. Gram-negative bacteria’s outer membrane is mostly made up of LPS, which binds to the Toll-like receptor 4 (*TLR4*). Toll-like receptor 3 (*TLR3*) is activated by Poly I:C, a synthetic analogue of double-stranded RNA commonly present in certain viruses (40). Both the synthetic viral RNA polyinosinic:polycytidylic acid (Poly I:C) and the bacterial endotoxin lipopolysaccharide (LPS) have potent innate immune responses and are frequently utilized in rats to imitate the pathogenic processes brought on by bacteria and viruses, respectively (41). Results demonstrated that *FerH* expression was significantly high in the ovaries, muscles, intestines, and gills of normal crayfish (**Figure 4**). Target gene expression levels were frequently downregulated during the initial stages of pathogen challenge. This phenomenon’s origin was attributed to the emergency response. There were, however, certain exceptions. After pathogens challenge, the mRNA transcript of *FerH* gene was upregulated significantly in the muscles, intestines, and gills of crayfish (**Figure 5**). We speculated that *FerH* gene was probably involved in pathogen defense mechanisms.

*In vivo* studies of the target gene’s physiological properties have been conducted in crustaceans using RNA interference (RNAi), which has proven to be a successful technique (42). For example, all of these results suggested that the Pc-traf6 like gene played a critical cytoplasmic adapter role in regulating the synthesis of downstream effectors in the crayfish TLR signaling cascade (29). Additionally, the results of RNAi-mediated TRAF6 knockdown in *C. gigas* showed that Cg-TRAF6 may have contributed to innate immunity defense against viruses and bacteria (43). After the *FerH* gene was knocked down in this work, the expression level of downstream genes in the immunological signaling pathway was considerably downregulated (**Figures 7**, **8**). We predicted that via controlling the activities of downstream molecules, the FerH gene affected or regulated a number of important innate immune responses.

## 5 Conclusion

Our previous transcriptome test allowed us to identify the PcFerH gene in *P. clarkii*. When crayfish were exposed to LPS or Poly I: C, their muscles, intestines, and gills all showed substantially increased expression levels. After *PcFerH* was knocked down, the levels of downstream genes in immunological signaling pathway were inhibited. Together, our research demonstrated the significance of the *PcFerH* gene as an immunological signaling protein and their importance in regulating the expression of downstream effectors in immunological signaling system of crayfish.

## Data availability statement

The original contributions presented in the study are included in the article/supplementary material. Further inquiries can be directed to the corresponding authors.

## Ethics statement

The manuscript presents research on animals that do not require ethical approval for their study.

## Author contributions

S-PZ: Formal analysis, Methodology, Writing – original draft. Q-NL: Funding acquisition, Project administration, Supervision, Writing – review & editing. JZ: Data curation, Methodology, Writing – original draft. Q-HW: Data curation, Formal analysis, Investigation, Writing – original draft. YY: Data curation, Formal analysis, Writing – original draft. D-ZZ: Resources, Writing – original draft. B-PT: Funding acquisition, Project administration, Supervision, Writing – review & editing. L-SD: Project administration, Supervision, Writing – review & editing.
